# Optimizing of particle accelerated rotor parameters using the discrete element method

**DOI:** 10.1038/s41598-023-46359-7

**Published:** 2023-11-01

**Authors:** Bo Sun, Shizhong Wei, Lu Yang, Peng Li, Shuaiwu Tong

**Affiliations:** 1 School of Mechatronics Engineering, Henan University of Science and Technology, Luoyang, 471003, Henan China; 2 College of Agricultural Equipment Engineering, Henan University of Science and Technology, Luoyang, 471003 Henan China; 3 Joint Engineering Research Center for Abrasion Control and Moulding of Metal Materials, Henan University of Science and Technology, Luoyang, 471003 Henan China; 4 School of Materials Science and Engineering, Henan University of Science and Technology, Luoyang, 471003 Henan China

**Keywords:** Mechanical engineering, Computational methods

## Abstract

The acceleration capability of a centrifugal jet rotor plays a crucial role in achieving a high injection velocity of powder particles in the centrifugal impact moulding process. In this regard, the focus of this article is on optimization of the runner shape. To this end, the lengths of the first and second acceleration sections (*L*_1_ and *L*_2_), and the angles between the first and second acceleration sections and between the second and third sections (*α*_1_ and *α*_2_) are considered as the rotor parameters. Simulations were conducted using multiple discrete elements to explore the influence of multiple input parameters on the response value, and a regression model was established between the parameters and the particle injection rate. The experimental results show that the selected parameters significantly affect the rate of particle injection, and the interactions between the parameters *L*_1_ and *L*_2_, and between *L*_2_ and *α*_2_ have the largest effects. The results reveal that applying the optimized parameters improves the particle injection speed by 7.85% when compared to the pre-optimization model. This improvement in the rotor acceleration provides the basis for improving the efficiency of centrifugal impact moulding of metal powders.

## Introduction

Studies show that accelerated powder particles impinging on a solid-state substrate can provide a coating on the substrate surface by producing a large plastic strain deposit^1–5^. In this technology, the velocity of the particles is the key factor in combining the particle with the substrate after impact. Currently, the common particle acceleration methods include the airflow acceleration method, mechanical acceleration method, electromagnetic acceleration method, ultrasonic acceleration method and electrostatic acceleration method. It is worth noting that each method has advantages and limitations and can be employed in various applications. In this context, the airflow acceleration method has attracted many researchers focusing on the optimization of jet structures to increase the acceleration capacity of particles. Wan^6^ studied the effect of the various parameters of the powder jet on the impact velocity and particle distribution and developed a mathematical hydrodynamic model. Gao^7^ proposed the degree of aggregation (PAD) concept and analysed the effects of the nozzle geometry on the powder flow. Cao^8^ designed the nozzle geometry based on the selected particles and propellant gases for ejected particles to reach the maximum impact velocity. Furthermore, Buhl^9^ established an analytical model, analysed the motion of the particles, and modified the nozzle structure based on CFD simulation results. Forero-Sossa^10^ investigated the effect of the nozzle geometry on the deposition of hydroxy-apatite particles in LPCS and demonstrated that the nozzle geometry affects the coating uniformity. Zavalan^11^ employed an objective multi-optimization method to optimize the nozzle structure and improve nozzle performance. Klinkov^12^ employed a cylindrical barrel with a double-edged bevelled exit vortex to generate a high-speed two-phase flow. This approach was suggested as an alternative to cold spray nozzles. To resolve the shortcomings of conventional DeLaval nozzles in the cold-air dynamic spraying process, Liao^13^ proposed a modified nozzle structure and verified its acceleration performance through numerical simulations. In all the aforementioned studies, it was observed that the internal structure of the flow channel exhibits a substantial influence on the acceleration effect of powder particles.

Based on the performed literature survey, the focus of present study is on investigating the mechanical acceleration of powder particles using centrifugal throw rotors. To this end, the acceleration process involving the throwing of rotors with variously shaped runners is simulated using EDEM discrete-element software. The main objective of this article is to analyse the influences of the runner control parameters on the injection velocity of particles, establish a model to numerically analyse the influencing factors, and optimize the control parameters of the runner to improve the rotor acceleration performance.

## Materials and methods

In this article, the discrete element method is chosen as the simulation technique. This selection is made primarily because the perturbation of the particle motion caused by the airflow is often neglected when analysing the effect of the flow channel structure on the particle acceleration performance^14–17^. The fundamental idea behind the discrete element method is to consider the medium as a discrete body. Then the structure is discretized into independent elements and particles, where each particle is assumed to Newton's second law of motion. In this approach, the macroscopic motion of the entire medium is calculated by iteratively solving the governing equation^18,19^. To simulate the particle motion using the discrete unitary method, vibrational equations of motion are used to model particle‒particle collisions and the motion of particles at boundaries^20,21^.

### Modelling particle accelerating rotors

**Figure 1:**
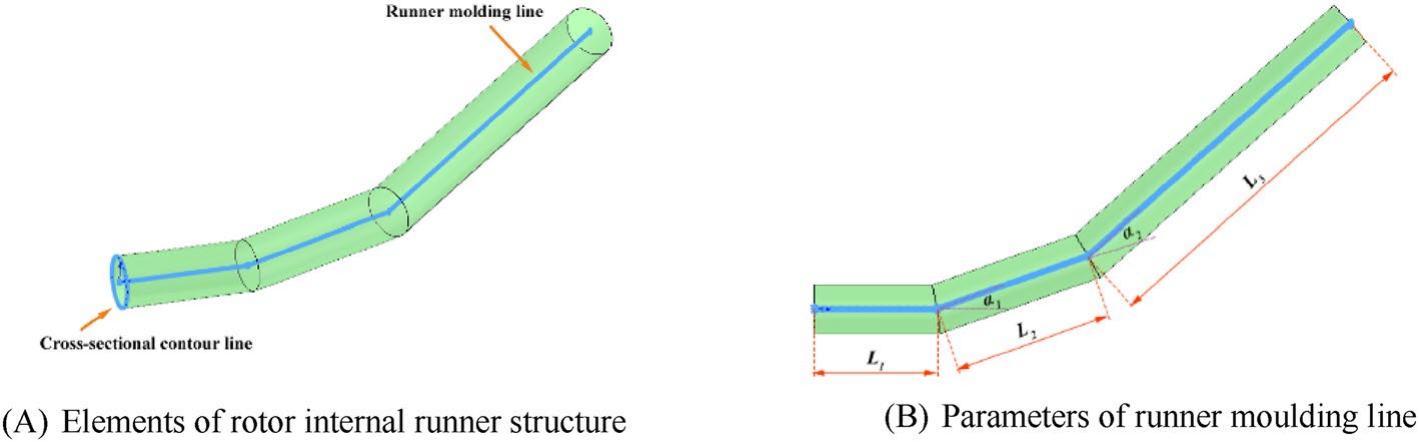
Rotor internal runner structure and its moulding control parameters. This figure consists of two plots. Two components of the runner inside the rotor are shown in Plot (**a**): the runner moulding line and the cross-section contour line. Moreover, the control parameters of the flow to the molding line are shown in Plot (**b**).

The rotor’s internal structure of the runner consists of two elements: the runner modelling line and the section contour line, as shown in Fig. 1A, the former determines the structural characteristics of the runner and the latter determines the cross-sectional shape of the runner .The runner modelling line is composed of multi-segment lines, and the following five parameters are implemented for line control: the lengths of the first acceleration section (*L*_1_), the lengths of the second acceleration section (*L*_2_), the lengths of the third acceleration section (*L*_3_), the angle between the first and second acceleration sections (*α*_1_) and the angle between the second and third accelerating sections (*α*_2_). These parameters, which are referred to as shape parameters, are shown in Fig. 1B. The parameter* L*_3_ can be calculated from *L*_1_, *L*_2_, *α*_1_, *α*_2_ and the known rotor radius (*R*) using Eq. (1). Therefore, the rotor optimization design is tested with the four key parameters *L*_1_, *L*_2_, *α*_1_, and *α*_2_ in a vehicle.

1 $$L_{3} = \sqrt {R^{2} - [\sin \left( {\alpha_{1} + \alpha_{2} } \right)L_{1} + \sin \alpha_{2} L_{2} ]^{2} } - \cos (\alpha_{1} + \alpha_{2} )L_{1} - \cos \alpha_{2} L_{2}$$

**Figure 2:**
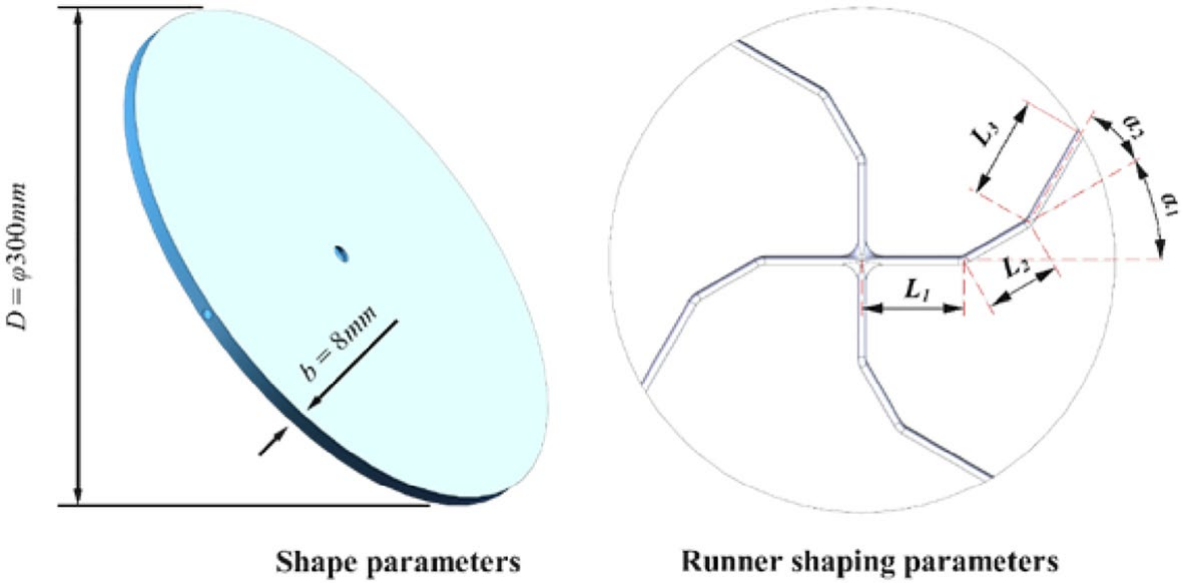
Rotor structure diagram. This figure shows the internal and external structure of the particle acceleration rotor.

The rotor is modelled using the SolidWorks software platform, and its internal and external structures are illustrated in Fig. 2. The parameter for the rotor are shown in Table 1, and the material properties are provided in Table 2.

**Table 1:** Table of the rotor model parameters.

Parameters	Value
Outer diameter of the rotor D/mm	300
Thickness of the rotor *b*/mm	8
Cross-sectional shape of the runner	Circle
Internal diameter of the runner section *d*/mm	5
Parameters of the rotor	*L*_1_, *L*_2_, *α*_1_, *α*_2_

**Table 2:** Table of rotor material parameters.

Parameters	Value
Density of the material kg∙m^3^	7.85 × 10^3^
Poisson's ratio of the material	0.3
Shear modulus of material MPa	2.06 × 10^5^

### Powder particulate model

Powder particles are usually spherical with smooth surfaces. To simplify the simulation, the hard-sphere model was employed in this study. The shape parameters and material properties are provided in Table 3.

**Table 3:** Pellet shape and material parameters.

Parameters	Value
Radius of the particle mm	0.5
Density of the material kg∙m^3^	2.7 × 10^3^
Poisson's ratio of the material	0.33
Shear modulus of the material MPa	6.89 × 10^3^

### Contact pattern

**Figure 3:**
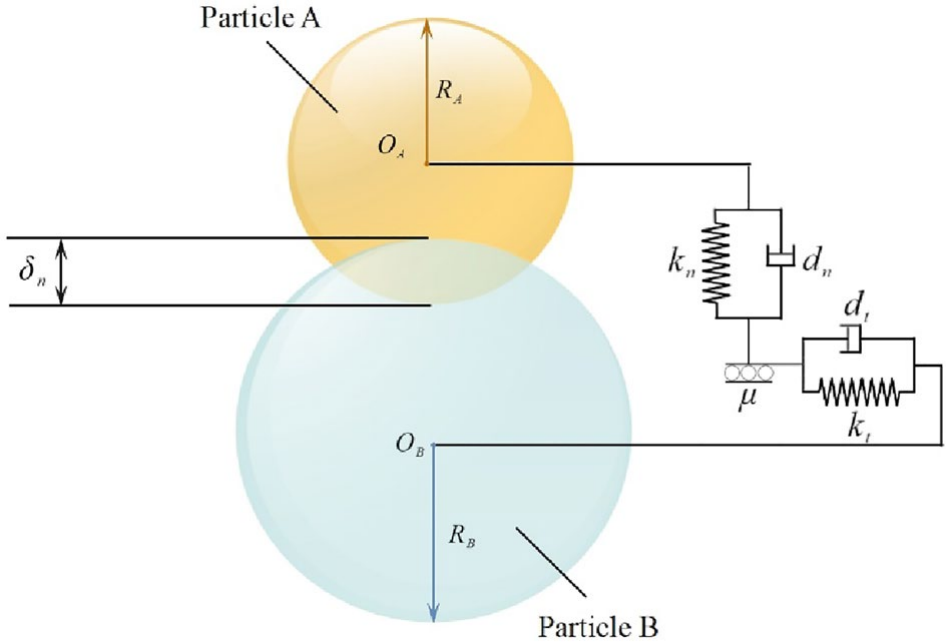
Hertz‒Mindlin no-slip contact mechanics model. This figure illustrates the Hertz‒Mindlin no-slip contact mechanics model.

Several contact models, including the Hertz‒Mindlin no-slip contact model, the Hertz‒Mindlin adhesion contact model, the linear adhesion contact model, the kinematic contact model at the surface, the linear elastic contact model, and the frictional loaded contact model, are commonly used^22^. Since the powder particles are spherical and there is no adhesion on their surfaces, the Hertz‒Mindlin no-slip contact model is selected to simulate particle‒particle and particle-rotor contacts. This model is schematically illustrated in Fig. 3. As shown in this figure, the contact forces between two particles fundamentally simplify into springs (with normal stiffness $k_{n}$ and tangential stiffness $k_{t}$), dampers (with normal damping $d_{n}$ and tangential damping $d_{t}$) and a slider (with friction coefficient $\mu$)^23^.

In this model, the normal force between the particles, tangential force, normal damping force, and tangential damping force can be calculated using the following equations:

2a $$F_{n} = \frac{4}{3}E^{*} \sqrt {R^{*} \alpha^{3} }$$

2b $$F_{t} = - S_{t} \delta$$

2c $$F_{n}^{d} = - 2\sqrt{\frac{5}{6}} \beta \sqrt {S_{n} m^{*} } \upsilon_{n}^{rel}$$

2d $$\frac{1}{{R^{*} }} = \frac{1}{{R_{A} }} + \frac{1}{{R_{B} }}$$

2e $$\frac{1}{{E^{*} }} = \frac{{1 - \nu_{A}^{2} }}{{E_{A} }} + \frac{{1 - \nu_{B}^{2} }}{{E_{B} }}$$

2f $$\alpha = R_{A} + R_{B} - \left| {{\mathbf{r}}_{A} - {\mathbf{r}}_{B} } \right|$$

2g $$S_{t} = 8G^{*} \sqrt {R^{*} \alpha }$$

2h $$S_{n} = 2E^{*} \sqrt {R^{*} \alpha }$$

2i $$\beta = \frac{\ln \varepsilon }{{\sqrt {\ln^{2} \varepsilon + \pi^{2} } }}$$

2j $$m^{*} = \frac{{m_{A} m_{B} }}{{m_{A} + m_{B} }}$$

2k $$\upsilon_{n}^{rel} = \left( {{{\varvec{\upupsilon}}}_{A} - {{\varvec{\upupsilon}}}_{B} } \right)n$$

2l $$G^{*} = \frac{{2 - \upsilon_{A}^{2} }}{{G_{A} }} + \frac{{2 - \upsilon_{B}^{2} }}{{G_{B} }}$$

2m $${\mathbf{n}} = \frac{{{\mathbf{r}}_{A} - {\mathbf{r}}_{B} }}{{\left| {{\mathbf{r}}_{A} - {\mathbf{r}}_{B} } \right|}}$$

2n $$T_{i} = - \mu_{r} F_{n} R_{i} \omega_{i}$$

The parameters in the above equation are defined as follows:

$E^{*}$ is the equivalent modulus of elasticity, Pa; $R^{*}$ is the equivalent particle radius, m; $\alpha$ is the normal overlap, m; $\delta$ is the tangential overlap, m; $S_{t}$ is the tangential stiffness, N/m; $S_{n}$ is the normal stiffness, N/m; $m^{*}$ is the equivalent mass, kg; $\upsilon_{n}^{rel}$ is the normal relative velocity, m/s; $\upsilon_{t}^{rel}$ is the tangential relative velocity, m/s; $\nu_{A}$,$\nu_{B}$ is the Poisson's ratio of the particles A&B; $E_{A}$,$E_{B}$ is the elastic modulus of the particle A&B, Pa; $R_{A}$,$R_{B}$ is the radius of the particle A&B, m; ${\mathbf{r}}_{A}$,${\mathbf{r}}_{B}$ is the spherical central position vector of the particle A&B, m; ${{\varvec{\upupsilon}}}_{A}$,${{\varvec{\upupsilon}}}_{B}$ is the velocity vector of the particles A&B before collision, m/s; ${\mathbf{n}}$ is the normal unit vector at the collision of the particles A&B; $G^{*}$ is the equivalent shear modulus, Pa; $\varepsilon$ is the recovery coefficient; $G_{A}$,$G_{B}$ is the shear modulus of the particle A&B, Pa; $\mu_{r}$ is the rolling friction factor; $R_{i}$ is the distance from the mass centre to the contact point, mm; ${{\varvec{\upomega}}}_{{\mathbf{i}}}$ is the Unit angular velocity vector of the object at the contact point, rad/s.

### Simulation parameters

**Figure 4:**
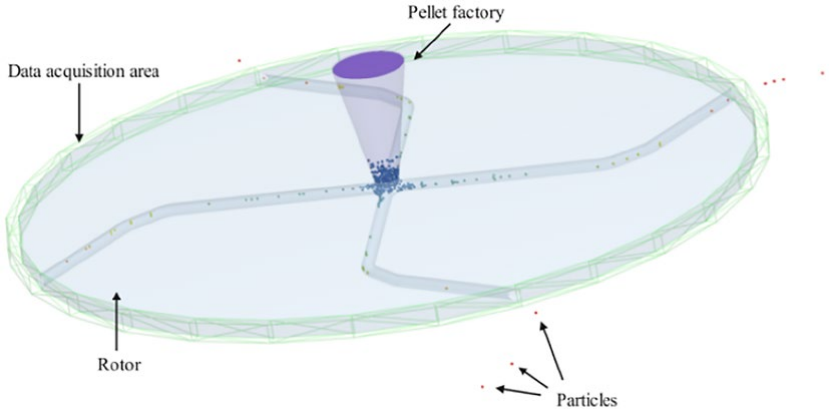
Simulation model. This figure illustrates the rotor-particle simulation modelling system utilized in this paper.

In the simulation, the rotor rotation speed is set to 30,000 rpm. A total of 5,000 powder particles were used in the analysis, and the simulation time was set to 0.05 s. Depending on the material of the rotor and particle, the coefficient of restitution, coefficient of static friction, and coefficient of rolling friction were selected as 0.1, 1.05, and 1.4, respectively. The Rayleigh wave method was utilized to determine the time step. Since the focus of this paper is on the launch speed and velocity of particles as they leave the rotor body, the annular grid area shown in Fig. 4 was used as the data acquisition area. In this area, is the instantaneous velocity of the particles can be determined as they are accelerated by the rotor. Figure 4 shows the configuration of the simulation model.

### Box‒Behnken design (BBD)

The BBD method is typically used to design response surface tests and perform an accurate statistical analysis of the test data to obtain an image with continuous features so that the mapping between the factors and the response values can be investigated visually^24^. In this article, a regression model needs to be developed using the particle ejection velocity* V* as the target quantity and the rotor parameters* L*_1_, *L*_2_, *α*_1_, and *α*_2_ as input factors. Then, the optimized design of the rotor can be achieved by solving the model. The use of the BBD response surface method allows complex unknown functional relationships between the target quantity and input factors to be fitted with simple primary or quadratic polynomial models over a small area, which is computationally easier and ensures the continuity of the predictive model. Using this method and performing ANOVA on their experimental data, this regression model can be obtained and solved for the optimal combination of parameters can be determined.

The empirical levels were determined based on the results obtained from the BBD tests shown in Table 4. Additionally, a preliminary analysis of the individual factors and a comprehensive consideration of their effects on acceleration was performed. To minimize measurement errors, each test was repeated three times and the mean values were recorded. The obtained results for each parameter level group are presented in Table 5.

**Table 4:** Factor ranges and levels.

Influence factors	Horizontal codes		
	−1	0	1
*L*_1_ (mm)	40	65	90
*L*_2_ (mm)	20	40	60
*α*_1_ (°)	30	40	50
*α*_2_ (°)	10	20	30

**Table 5:** BBD experimental design and results.

No	*L*_1_ (mm)	*L*_2_ (mm)	*α*_1_ (°)	*α*_2_ (°)	*V *(m/s)	Standard deviation
1	90	60	40	20	577.67	0.130
2	40	20	40	20	575.55	0.124
3	90	20	40	20	567.87	0.093
4	65	40	40	20	578.67	0.120
5	40	40	50	20	574.86	0.139
6	90	40	40	10	579.76	0.131
7	90	40	40	30	555.30	0.128
8	40	40	40	30	575.65	0.104
9	65	40	40	20	575.59	0.086
10	90	40	50	20	557.34	0.071
11	65	20	40	10	575.63	0.149
12	90	40	30	20	582.47	0.094
13	65	40	40	20	576.25	0.078
14	65	40	30	10	578.06	0.129
15	65	60	40	10	582.64	0.080
16	65	40	50	10	576.01	0.086
17	40	40	40	10	572.19	0.104
18	65	40	30	30	573.73	0.107
19	65	60	50	20	573.42	0.106
20	40	40	30	20	574.32	0.099
21	40	60	40	20	577.89	0.093
22	65	60	40	30	568.16	0.108
23	65	40	50	30	559.46	0.094
24	65	20	30	20	577.66	0.106
25	65	20	50	20	570.65	0.127
26	65	40	40	20	575.98	0.088
27	65	20	40	30	570.35	0.062
28	65	40	40	20	577.02	0.099
29	65	60	30	20	582.44	0.115

## Results

Based on the results of ANOVA presented in Table 6, the obtained P-value for the regression model is less than 0.0001, indicating that the regression model is highly significant. In the meantime, the obtained P-value for the model is more than 0.05, indicating that the model misfit is not significant and that the regression model fit is high. Furthermore, based on the contents of Table 7, the *R*^2^ and adjusted* R*^2^ values in the model are all close to one, indicating a good fit of the regression model. The predicted *R*^2^ reasonably agrees with the adjusted *R*^2^, with a difference of less than 0.2. The coefficient of variation and precision are 0.25% and 27.23, respectively, indicating that the fitted regression model has a high degree of reliability.

**Table 6:** Analysis of the variance for model.

_Source of variance_	_Mean square_	_Degrees of freedom_	_Sum of squares_	_Value of F_	_Value of P_
_Model_	1274.49	14	91.03	45.41	< 0.0001
A-*L*_1_	75.25	1	75.25	37.54	< 0.0001
B-*L*_2_	50.06	1	50.06	24.97	0.0002
C-*α*_1_	270.18	1	270.18	134.77	< 0.0001
D-*α*_2_	316.62	1	316.62	157.94	< 0.0001
AB	13.91	1	13.91	6.94	0.0196
AC	164.74	1	164.74	82.17	< 0.0001
AD	194.88	1	194.88	97.21	< 0.0001
BC	1.01	1	1.01	0.5038	0.4895
BD	21.16	1	21.16	10.55	0.0058
CD	37.33	1	37.33	18.62	0.0007
_A2_	50.39	1	50.39	25.14	0.0002
_B2_	4.63	1	4.63	2.31	0.1507
_C2_	16.47	1	16.47	8.22	0.0124
_D2_	69.72	1	69.72	34.78	< 0.0001
_Residual_	28.07	14	2		
_Lack of fit_	22.13	10	2.21	1.49	0.3729
_Pure error_	5.94	4	1.48		
_Cor total_	1302.56	28

**Table 7:** Fit statistics.

Statistical indicator	Value
*R*2	0.9785
Adjusted *R*2	0.9569
Predicted *R*2	0.8950
Coefficient of variation (C.V.%)	0.2467
Adeq precision	27.2340

The P-values in Table 6 for the parameters* L*_1_, *L*_2_, *α*_1_, and *α*_2_ indicate that all four test factors have a highly significant effect on the injection velocity of particles. Among them, the effects of *L*_1_, *α*_1_, and *α*_2_ are more significant compared to that of *L*_2_. The presented results in Table 6 obtained from the one-way ANOVA confirm that the parameters *L*_1_, *α*_1_, and *α*_2_ significantly affect the particle injection rate, while the effect of the parameter *L*_2_ is relatively smaller.

The parameters *L*_1_, *L*_2_, *α*_1_, and *α*_2_ were selected as independent variables and denoted as *x*_1_, *x*_2_, *x*_3_, and *x*_4_, respectively. Moreover, the injection velocity of particles, denoted as *V*, was selected as the target variable. The least-squares method was employed to optimize the regression model. In the optimization process, four groups of single-factor first-order terms, four groups of single-factor second-order terms, and six groups of interactive-factor second-order terms were considered. Since the P-value reflects the significance of each factor in the model, when P ≤ 0.01, the factor is considered highly significant; when 0.01 < P < 0.05, the factor is significant; and when P ≥ 0.05, the factor is not significant. The model can be optimized according to the P-value of each factor. Consequently, the terms BC (interaction terms for *L*_2_ and *α*_1_) and B^2^ (the quadratic term for *L*_2_) with P > 0.05 were excluded from the model. The regression model for the injection rate *V* can be expressed as follows:

3 $$\begin{aligned} V & = 420.22 + 1.9488x_{1} + 0.0897x_{2} + 3.2078x_{3} + 4.3585x_{4} \\ & \;\;\; + 0.0037x_{1} x_{2} - 0.0257x_{1} x_{3} - 0.0279x_{1} x_{4} - 0.0115x_{2} x_{4} - 0.0306x_{3} x_{4} \\ & \;\;\; - 0.0047x_{1}^{2} - 0.0175x_{3}^{2} - 0.0344x_{4}^{2} \\ \end{aligned}$$

## Discussion

### The influence of the runners' shape parameters on the injection rate

#### The effect of the individual parameters on the injection velocity

**Figure 5:**
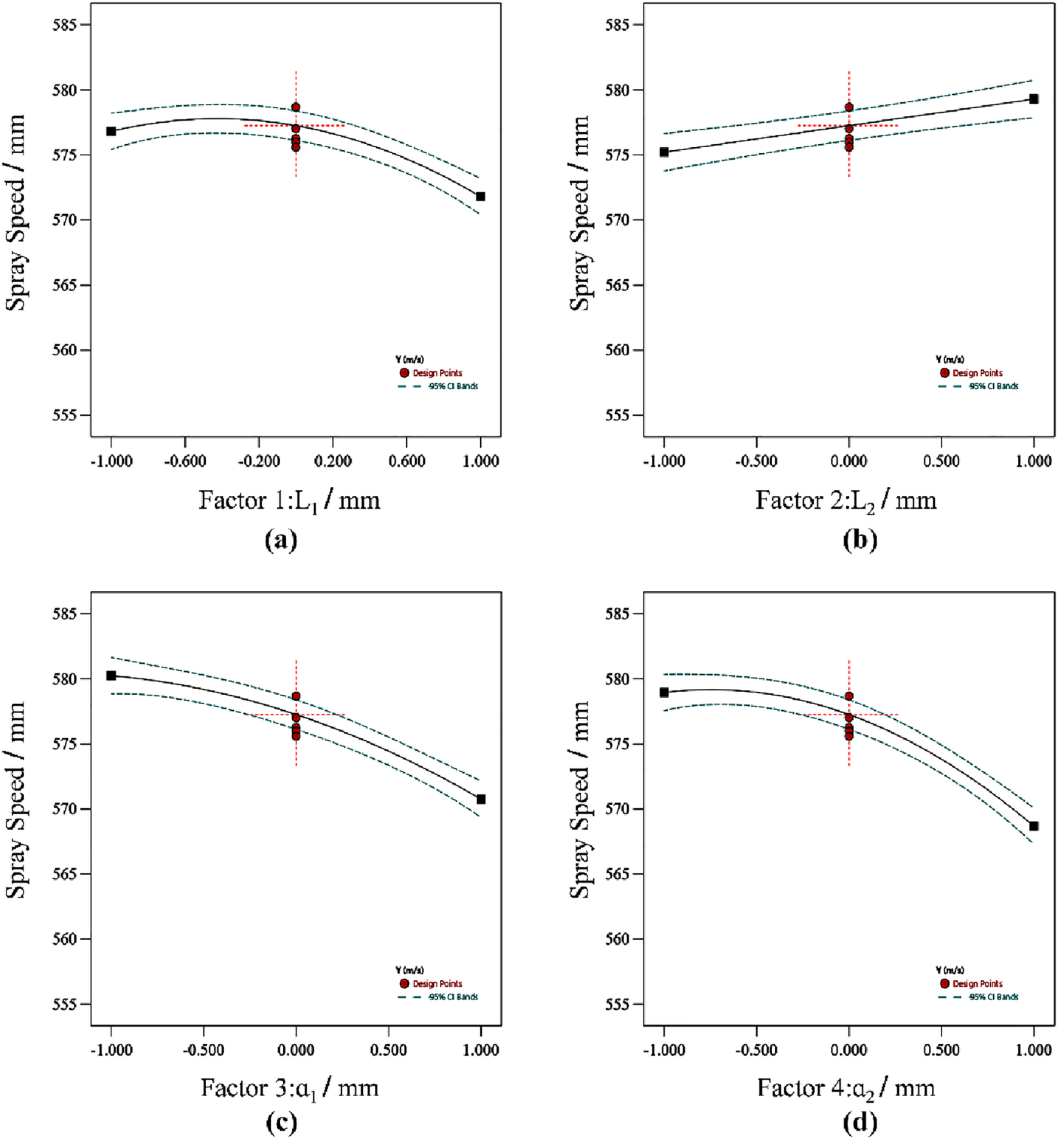
Effects of the individual parameters on the injection speed. This figure illustrates the influence of the multi-model parameter interactions on the injection rate. Plot (**a**) shows the impact of *L*_1_ on the injection rate while maintaining *L*_2_, *α*_1_, and *α*_2_ at their median values. Plot (**b**) shows the impact of *L*_2_ on the injection rate while maintaining *L*_1_, *α*_1_, and *α*_2_ at their median values. Plot (**c**) shows the impact of *α*_1_ on the injection rate while maintaining *L*_1_, *L*_2_, and *α*_2_ at their median values. Plot (**d**) shows the impact of *α*_2_ on the injection rate while maintaining *L*_1_, *L*_2_, and *α*_1_ at their median values.

Figure 5 provides insights into the influence of individual design parameters on the injection velocity. Figure 5a depicts the effect of *L*_1_ on the injection rate when *L*_2_, *α*_1_, and *α*_2_ are held constant at their median values. Similarly, Fig. 5b shows the impact of *L*_2_ on the injection rate while maintaining *L*_1_, *α*_1_, and *α*_2_ at their median values. Figure 5c demonstrates the influence of α_1_ on the injection rate with *L*_1_, *L*_2_, and *α*_2_ set at their median values. Finally, Fig. 5d illustrates the effect of *α*_2_ on injection velocity when* L*_1_,* L*_2_, and *α*_1_ are held constant at their median values.

In general, the design parameters *L*_1_, *α*_1_, and *α*_2_ exhibit a negative correlation with the injection velocity, while the parameter *L*_2_ shows a positive correlation. The trend for each parameter can be described as follows:

- As the parameter *L*_1_ increases from 40 to 60 mm, the injection velocity increases slightly. However, when *L*_1_ exceeds 60 mm, the ejecta velocity decreases.
- When the parameter* L*_2_ varies in the range of 20 mm to 60 mm, there is a positive correlation between *L*_2_ and the ejection velocity.
- When the design parameters *α*_1_ and *α*_2_ increase from 30 to 50 and from 10 to 30, respectively, the ejection velocity decreases.

The results reveal that *L*_1_, *L*_2_, *α*_1_, and *α*_2_ affect the particle injection velocity. The optimized design involves setting the parameter *L*_1_ around the median value (60 mm), selecting the maximal value of 60 mm for *L*_2_, and choosing the minimum values (30 and 10) for *α*_1_ and *α*_2_, respectively. Meanwhile, it is necessary to analyse the interaction between various factors.

#### The influence of the multi-model parameter interactions on the injection rate

Design-Expert13 software was utilized to generate 3D response surface plots depicting the interaction effects of each shape parameter. The results align with those of the ANOVA analysis: $P_{{L_{1} \alpha_{2} }} (0.0001)$ < $P_{{L_{1} \alpha_{1} }} (0.0001)$ < $P_{{\alpha_{1} \alpha_{2} }} (0.0007)$ < $P_{{L_{2} \alpha_{2} }} (0.0058)$ < $P_{{L_{1} L_{2} }} (0.0196)$. The interaction between the* L*_1_ and *L*_2_ parameters exhibits the most substantial impact on the particle injection velocity among the various parameter interactions. Then the interaction between the parameters *L*_2_ and *α*_2_ has a comparatively smaller effect, while the interaction between the parameters *α*_1_ and *α*_2_ has a much smaller effect. Last, the interactions between the parameters *L*_2_ and parameters *α*_2_ and *L*_2_ and *α*_2_ exhibit the weakest effects. The interaction effects of the pair of parameters *L*_1_ and *L*_2_, *L*_2_ and *α*_2_, and *α*_1_ and *α*_2_ are analysed.

**Figure 6:**
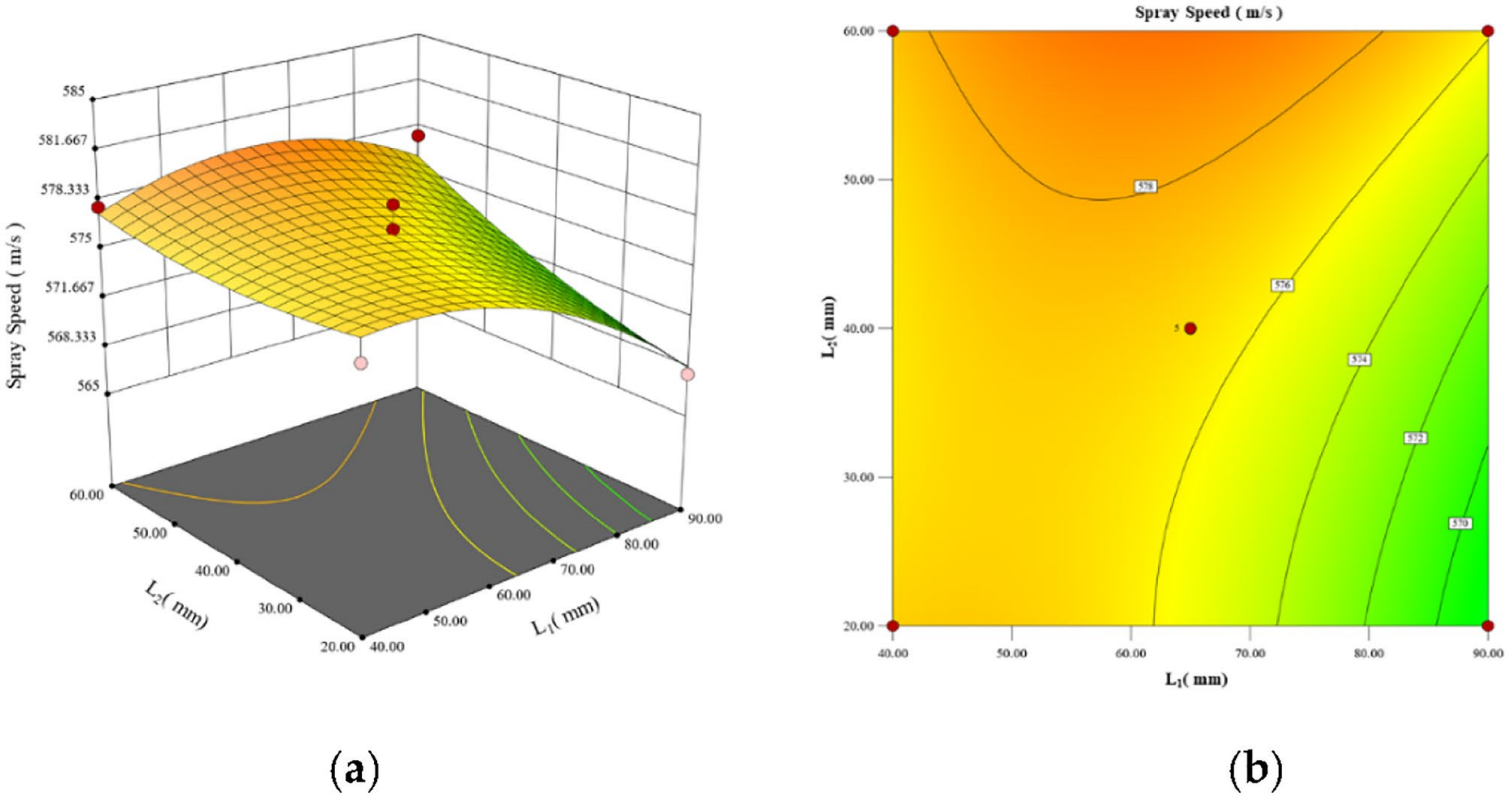
Mapping of the interaction effects of the parameters *L*_1_ and *L*_2_. This figure consists of two pictures. Plot (**a**) shows the response surface of the parameters *L*_1_ and *L*_2_, and Plot (**b**) shows the contour plots of the parameters *L*_1_ and *L*_2_, both of which reflect the interaction between the two parameters.

The interaction between the parameters *L*_1_ (ranging from 40 to 90 mm) and *L*_2_ (ranging from 20 to 60 mm) is illustrated in Fig. 6a, b. It is observed that as the values of *L*_1_ increase and the values of *L*_2_ decrease, the contour lines become along the 135 contour. This indicates a decrease in the jet velocity, which gradually transitions to an acceleration effect. Based on the distribution of the contours, the entire response surface has a maximum point of approximately 70 mm for *L*_1_ and 50 mm for *L*_2_. This implies that the length of each acceleration section should be relatively constant in a multi-segment model and that the difference in length should be minimized. For example, when *L*_1_ = 90 mm and *L*_2_ = 20 mm, the particle jet velocity reaches its minimum value.

**Figure 7:**
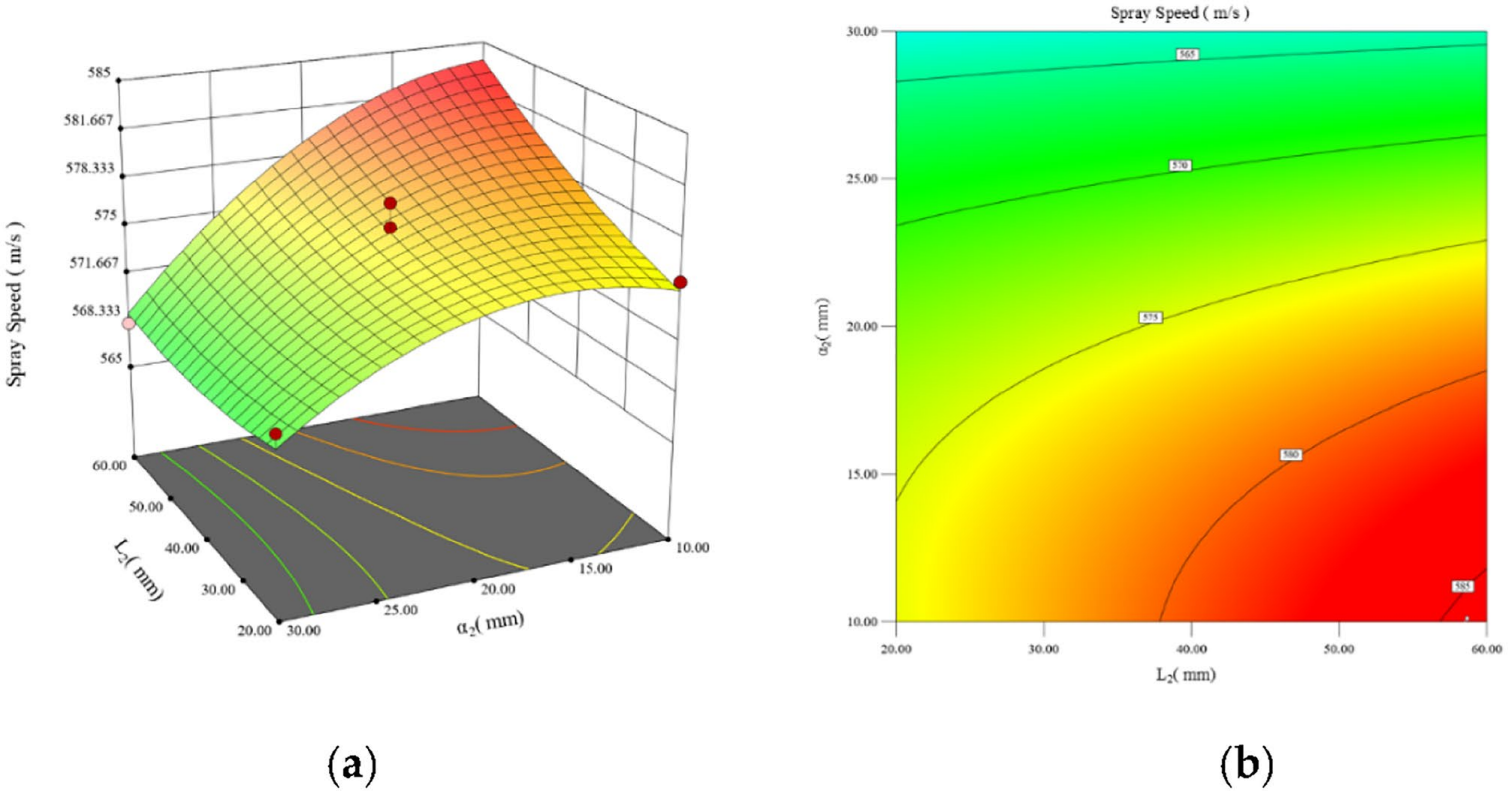
Mapping of the interaction effects of the parameters *L*_2_ and *α*_2_. This figure consists of two plots. Plot (**a**) shows the response surface of the parameters *L*_2_ and *α*_2_, and Plot (**b**) shows the contour plots of the parameters *L*_2_ and *α*_2_, both of which reflect the interaction between the two parameters.

Figure 7a, b illustrate the interaction effect of the parameters* L*_2_ (20 mm to 60 mm) and *α*_2_ (10–30). It is observed that for the parameter *L*_2_ with a value of 60 mm and *α*_2_ with a value of 10, the peak of the entire response surface is at the top right. Furthermore, the contours gradually concentrate in the direction of the lower right-hand corner, and the degree of curvature increases gradually indicating that as the value of *α*_2_ is increased from 30 to 10, the effect on the *L*_2_ increases gradually. This result demonstrates that a too-large angle between the acceleration sections affects the overall acceleration effect of the rotor.

**Figure 8:**
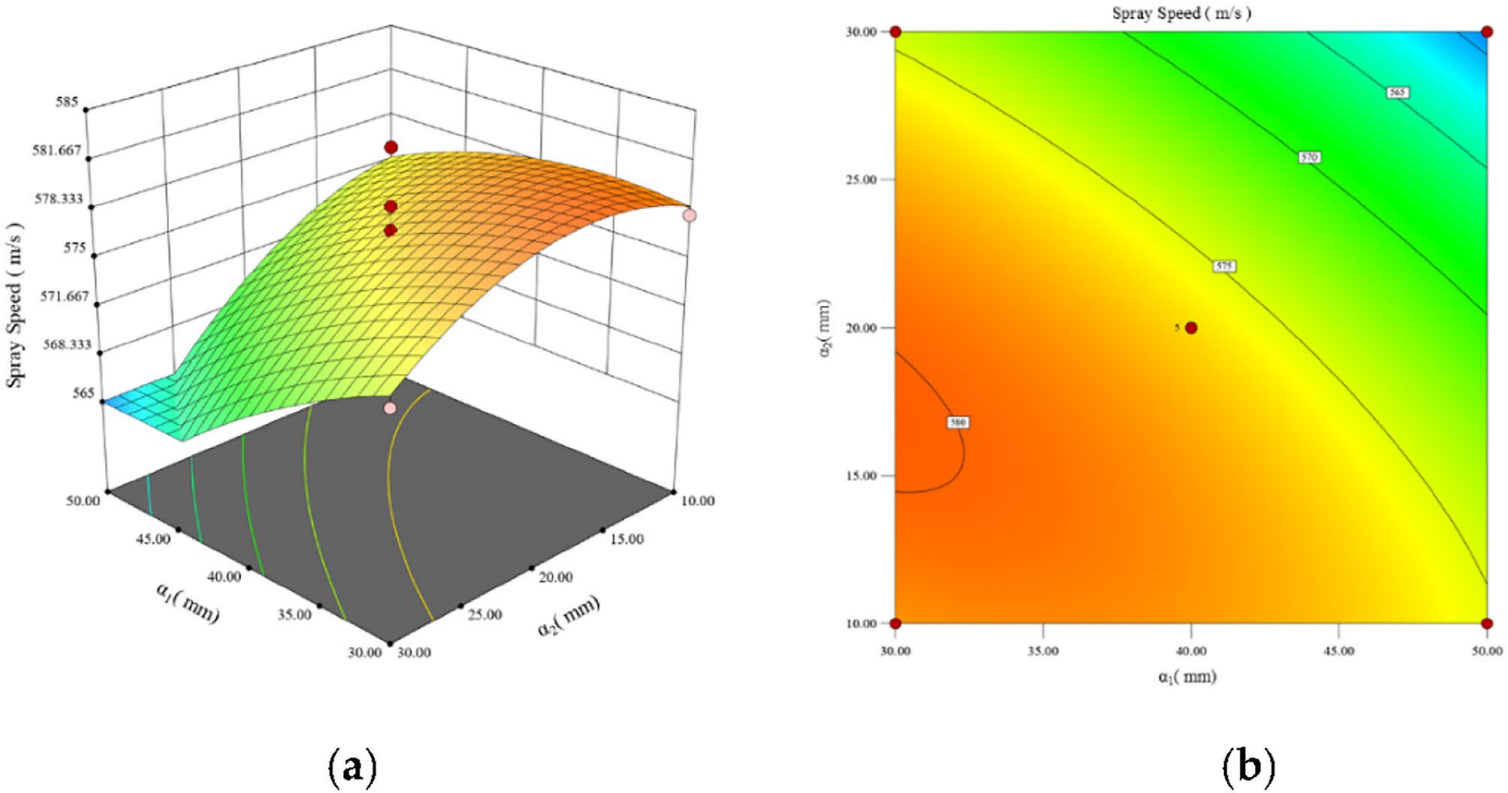
Mapping of the interaction effects of the parameters *α*_1_ and *α*_2_. This figure consists of two plots. Plot (**a**) shows the response surface of the parameters *α*_1_ and *α*_2_, and Plot (**b**) shows the contour plots of the parameters *α*_1_ and *α*_2_, both of which reflect the interaction between the two parameters.

Figure 8a, b illustrate the interaction analysis of the parameters *α*_1_ and *α*_2_. When the parameters *α*_1_ and *α*_2_ vary in the range of 30 to 40 and 10 to 20, respectively, the value of the response face has a high level of significance. The results also show a decreasing contour that thickens along 45, indicating that the particle jet velocity drops significantly as large values are selected for the parameters *α*_1_ and *α*_2_. This finding further confirms that the angle between the accelerating sections should not be too large.

Through the discussion of the independent effect of a single parameter and the interaction of multiple parameters on the spraying speed, we can conclude that the internal flow channel structure of the rotor has an important influence on the acceleration process of particles in which there exists a quantitative relationship between the structural parameters and the particle spraying speed (the acceleration capacity of the particles), which can be solved through the establishment of a mathematical model of this relationship. This change in acceleration is characterized by the acceleration process of powder particles in cold spraying^25–29^, which can be achieved by changing the nozzle structure to increase the acceleration capacity. However, due to the lack of analysis of the overall force on the acceleration process of individual particles in the current study, it is not yet possible to completely correspond the change in the runner structure to the acceleration process of the particles, and therefore, the current acquisition of the mathematical model is still based on the statistical foundations.

### Optimum parameters and verification

**Figure 9:**
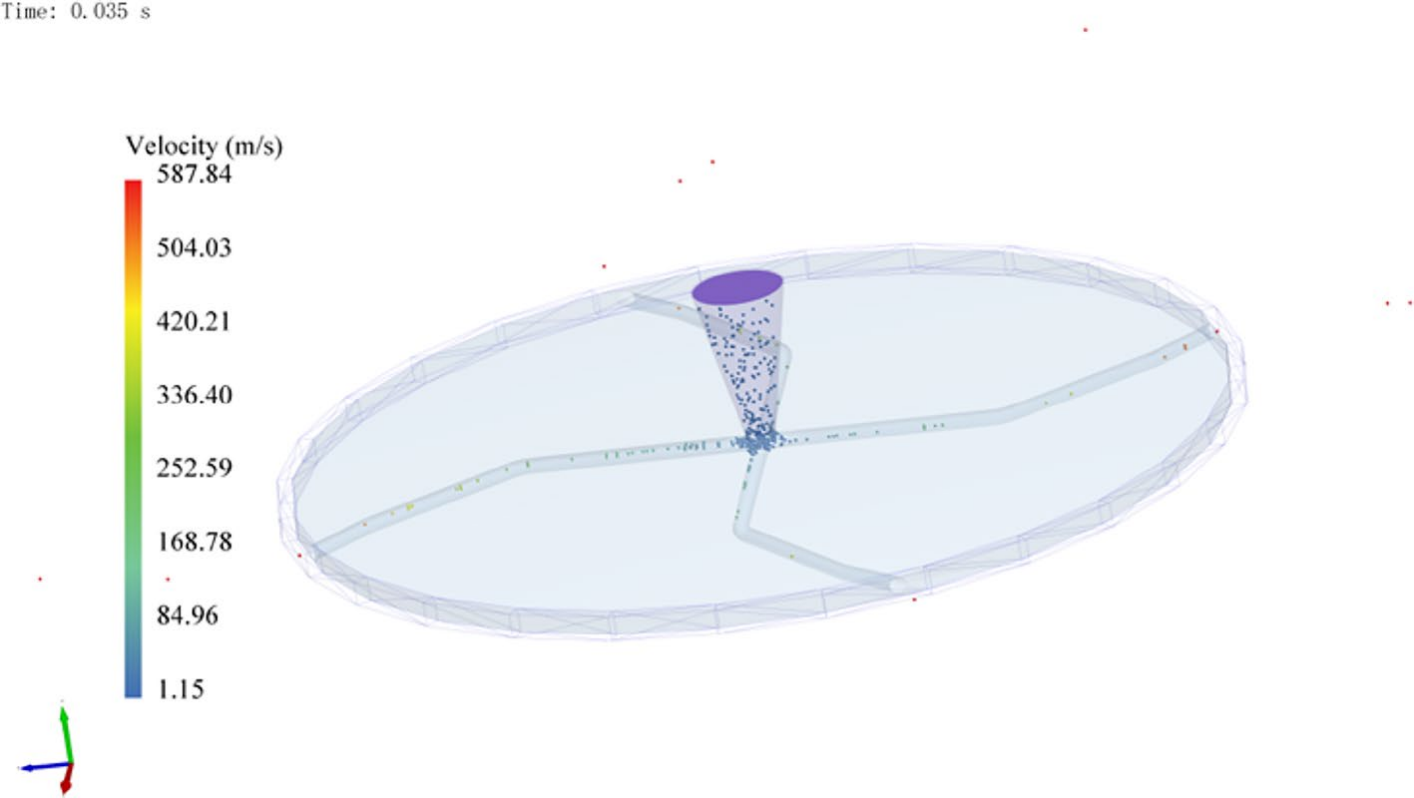
Acceleration of particles by the rotor after parameters optimization. This figure illustrates the acceleration of the particles by the rotor after shape parameter optimization.

**Figure 10:**
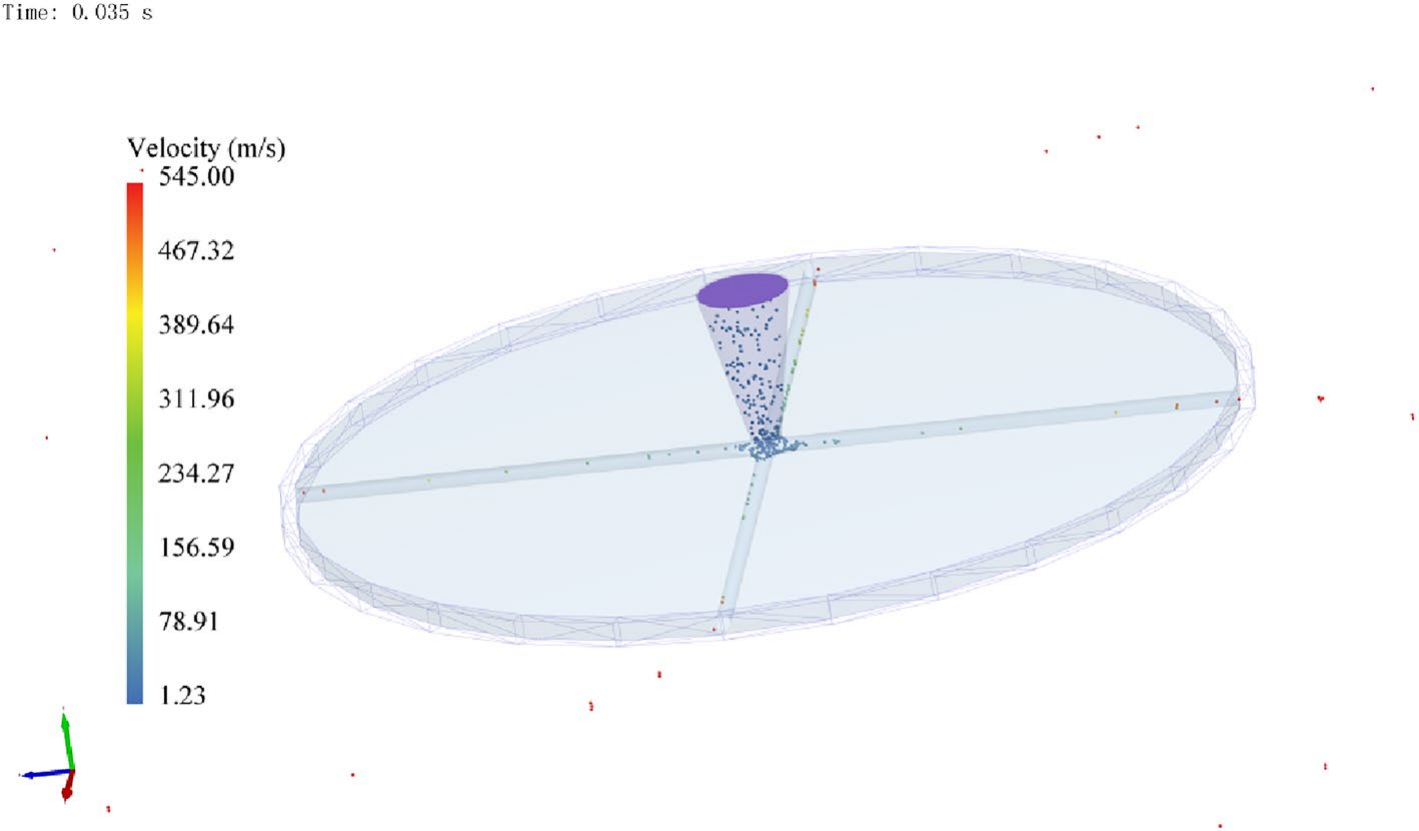
Acceleration of particles by the rotor with a through-hole runner. This figure illustrates the acceleration of the particles by the rotor with a through-hole runner.

Based on the solution of the regression model with the injection velocity as a condition, the optimal parameters are *L*_1_ = 77.37, *L*_2_ = 58.67, *α*_1_ = 39.74, and *α*_2_ = 10.11. The regression prediction of the injection velocity based on these parameters was 585.439 m/s, as illustrated in Fig. 9. Subsequently, using these optimal parameters to model the throwing rotor and conducting EDEM simulation tests, a jet speed of 587.841 was obtained, with a 0.408% error compared to the model prediction. This indicates that the established regression model can effectively predict the velocity of the particle jet based on the rotor runner parameters. Simultaneously, the EDEM simulation test of the rotor with a through-hole runner showed a particle injection speed of 545.00 m/s as illustrated in Fig. 10. By optimizing the runner design, the particle injection speed was increased by 7.85%, demonstrating that the capability of the jet rotor can be improved through runner optimization.

## Conclusions

In this paper, the particle accelerating rotor was used as a research object to investigate the effect of rotor parameters on its accelerating performance. Simulations of the particle acceleration by rotors with different parameters were conducted based on the discrete cell method. In addition, the rotor acceleration performances before and after parameter optimization were compared. The main conclusions can be summarized as follows.

1. In this study, the accelerated rotor is modelled using SolidWorks and its acceleration performance is simulated using EDEM. By analysing the simulation results of 25 groups of rotors, a multivariate nonlinear regression model is established for the first time based on rotor parameters.
2. By analysing the regression model, it is proven that all four modelling parameters have significant effects on the particle ejection velocity. It was found that *L*_1_, *α*_1_, and *α*_2_ are negatively correlated with the injection speed, while *L*_2_ exhibits a positive correlation. The interaction between *L*_1_ and *L*_2_ had the largest effect, followed by the interaction between* L*_2_ and *α*_2_, whereas the interaction between *α*_1_ and *α*_2_ had a smaller effect, and the interaction of the other factors was not significant.
3. The accelerating rotor parameters were optimized using a regression model for parametric optimization. By analysing the simulation results, the improvement in the rotor acceleration capacity before and after optimization is 7.85%.

The results show that the optimization of the rotor parameters can enhance the acceleration capacity for particles, which provides a feasible solution for improving the quality and efficiency of the centrifugal impact caused by metal powders. However, further improvement of this forming method requires in-depth studies of more complex rotor structures and forming environmental conditions, as well as the optimization of the forming process control strategies.

## Data Availability

The datasets generated during and/or analysed during the current study are available from the corresponding author upon reasonable request.
